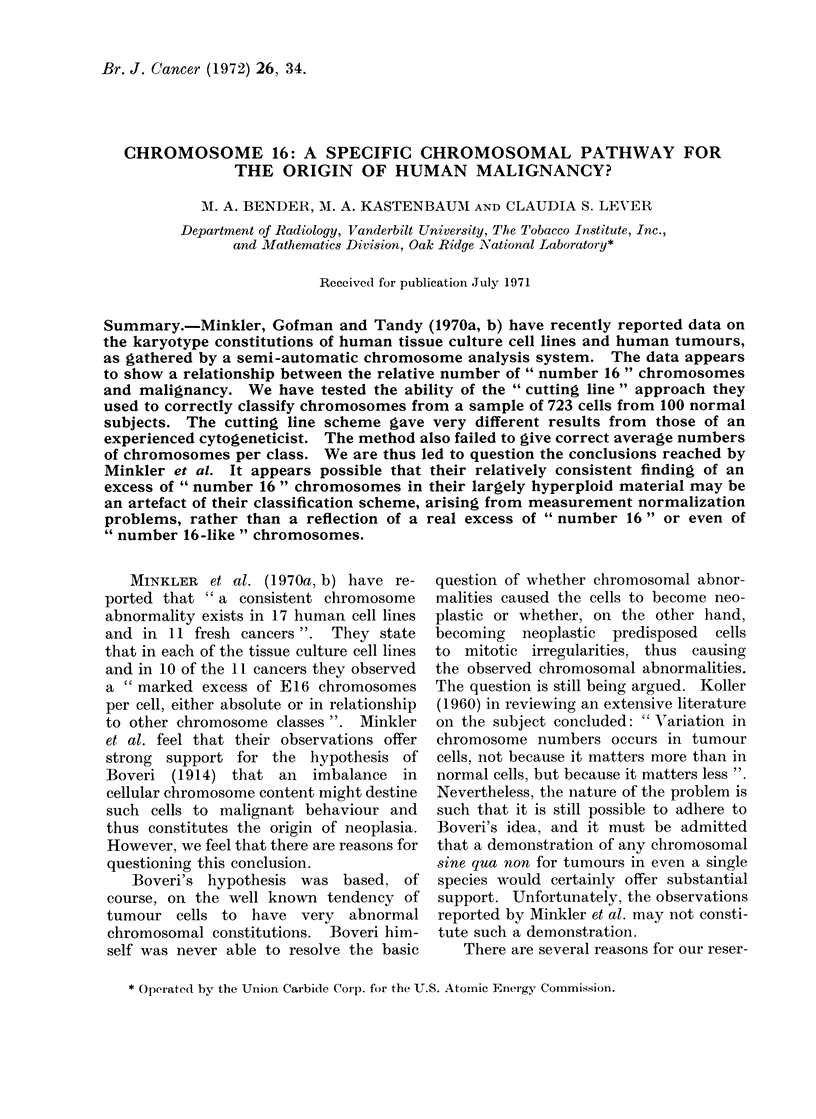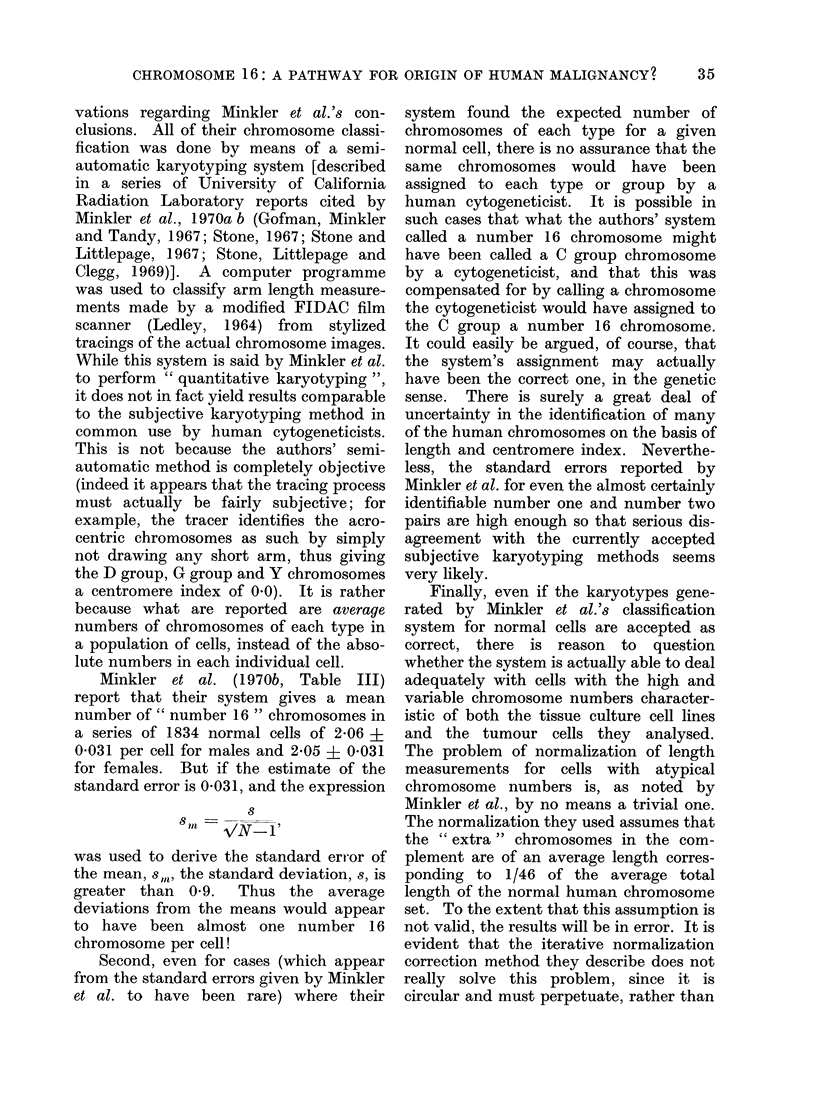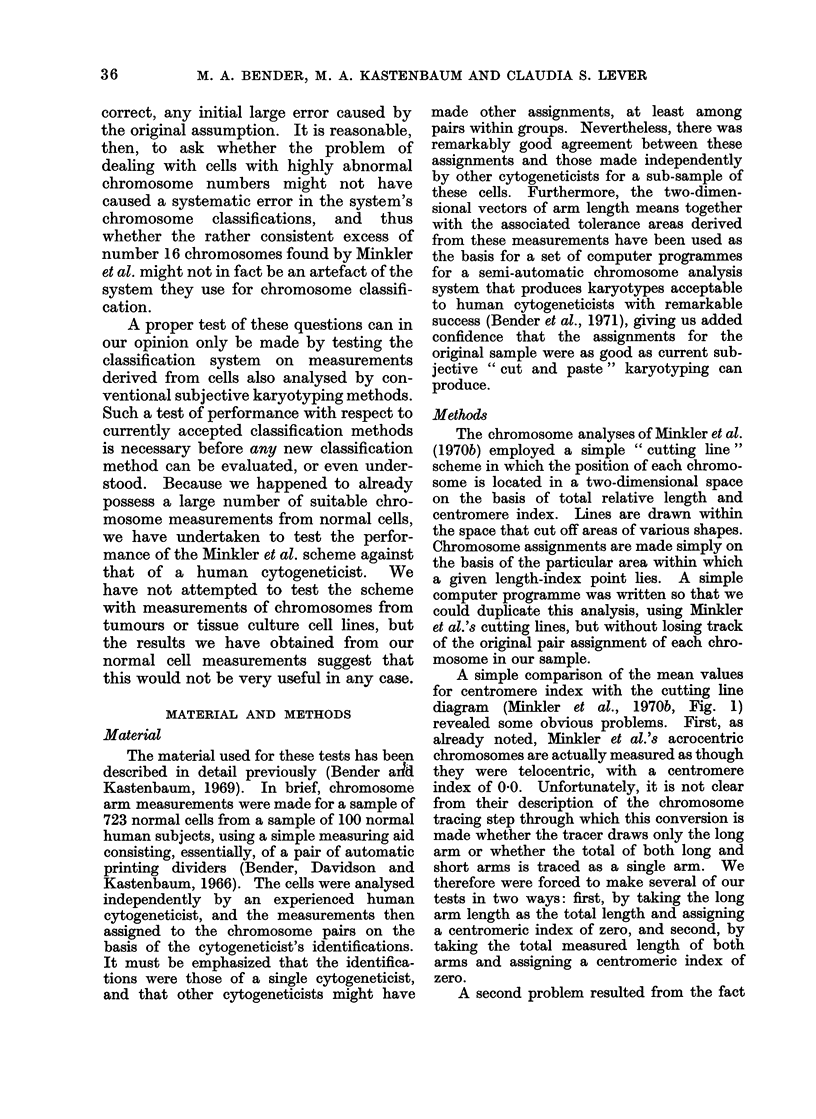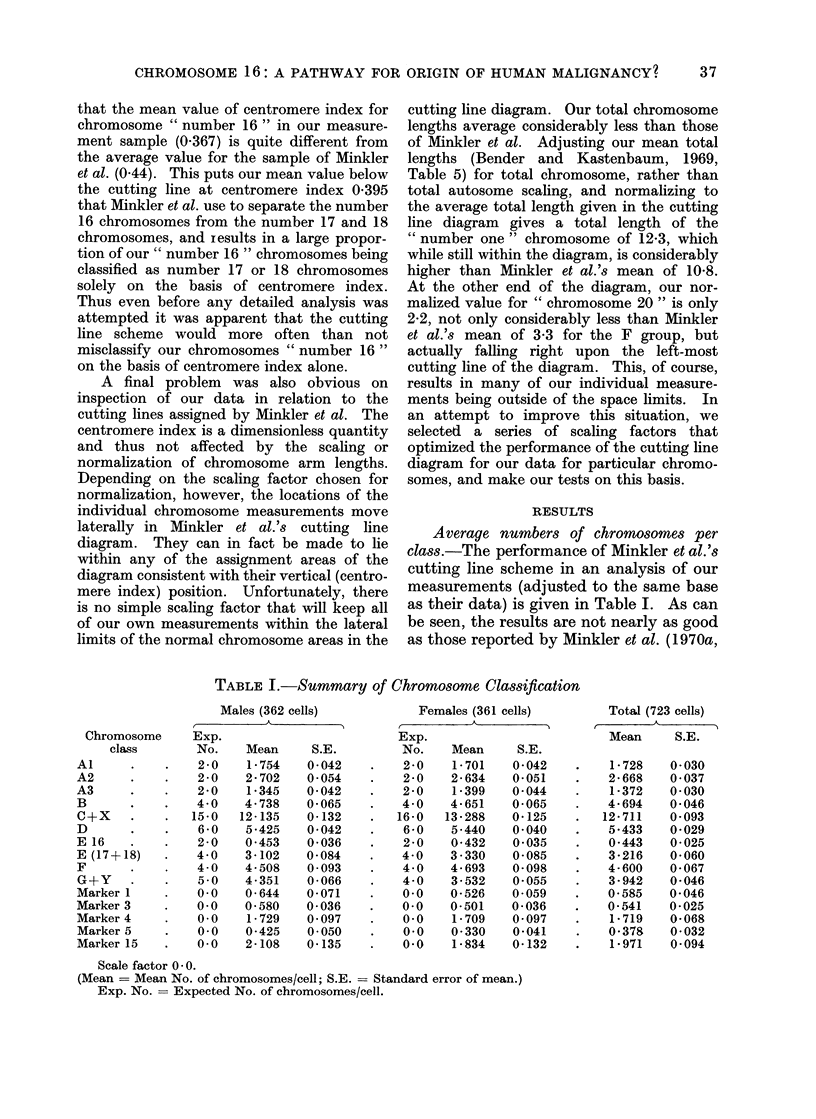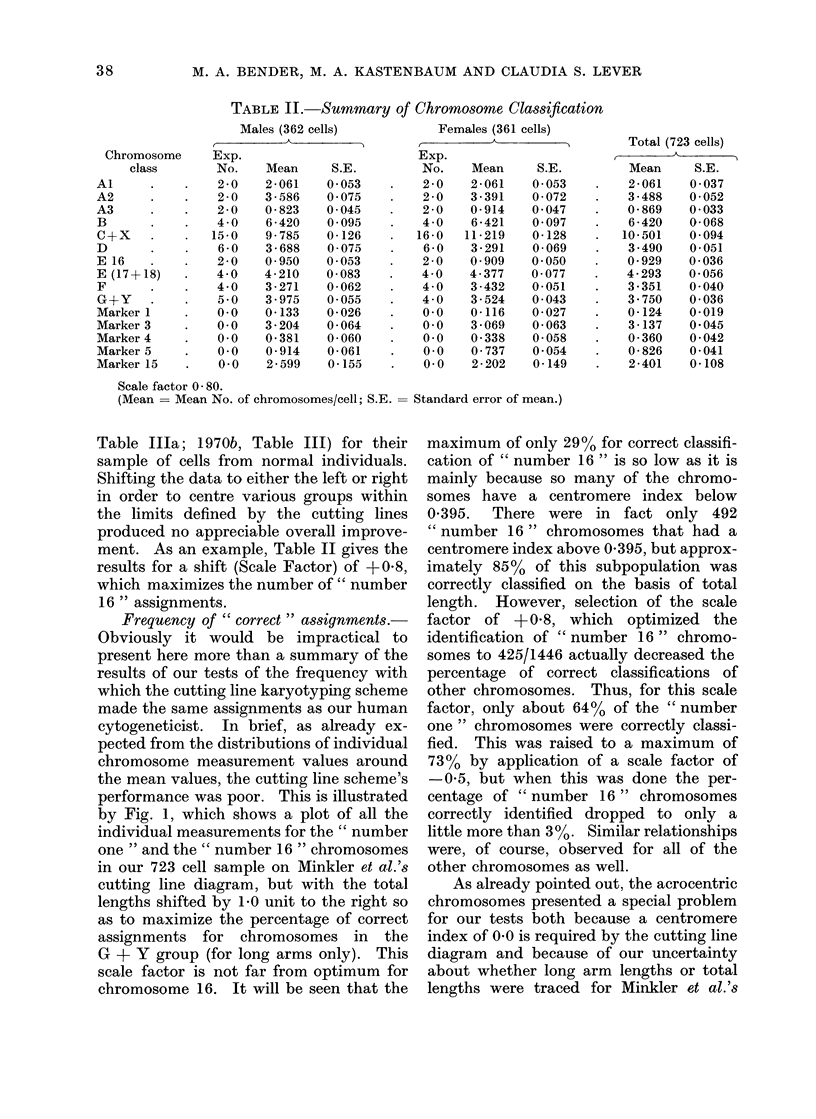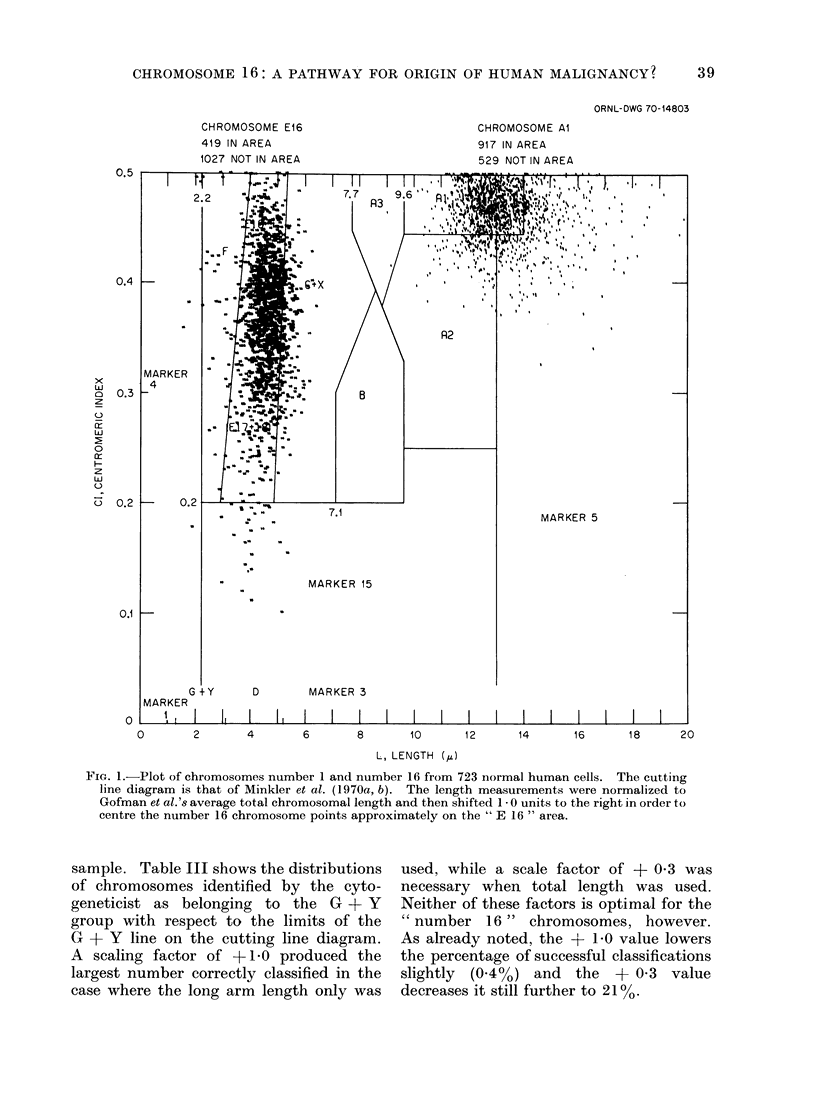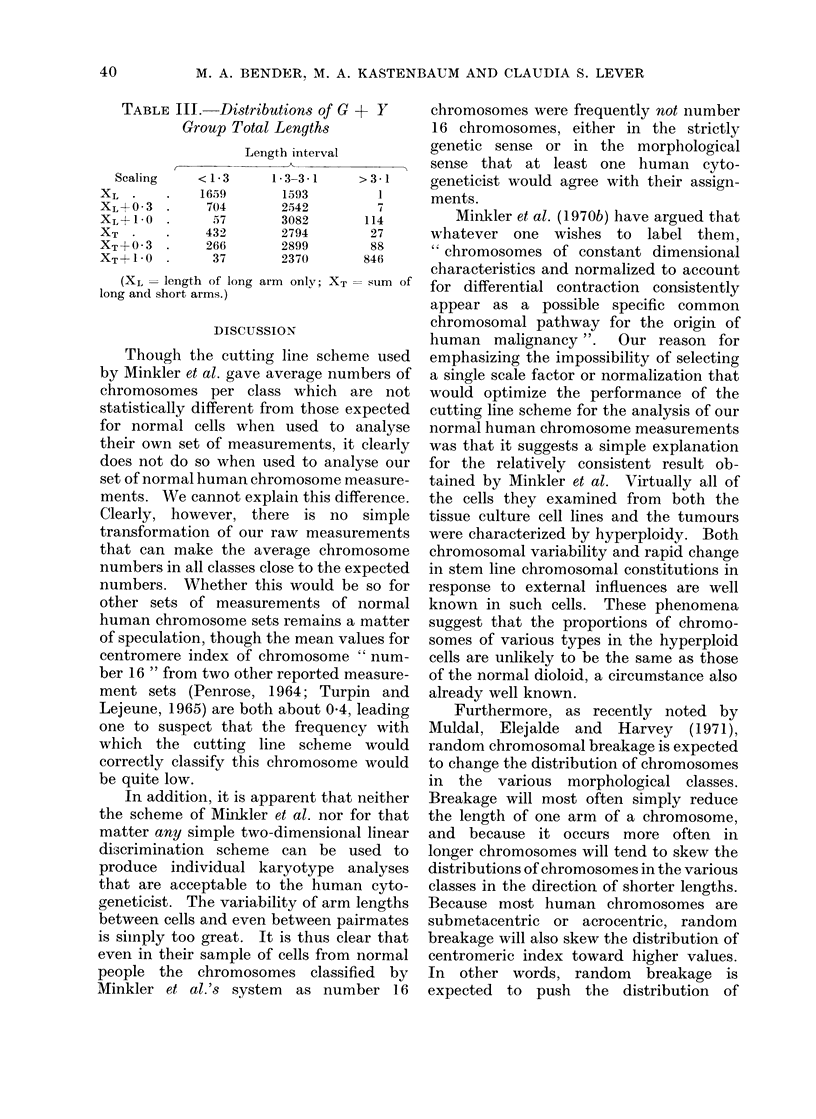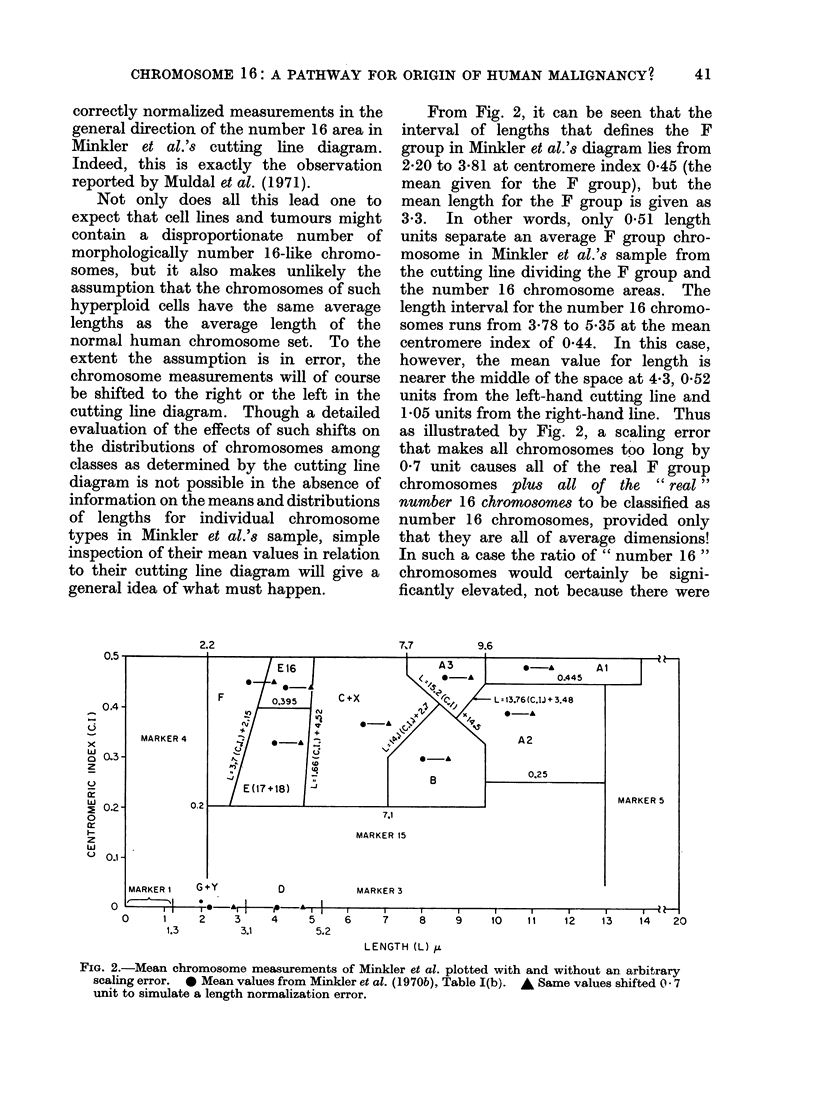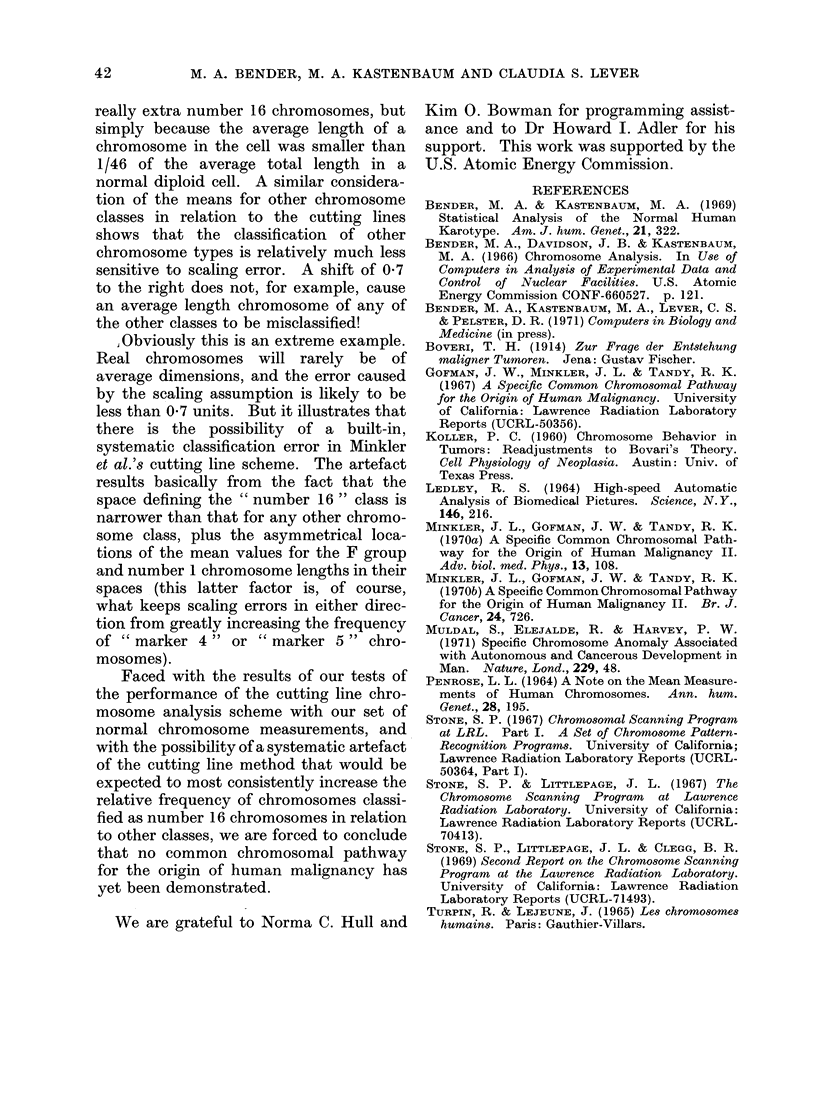# Chromosome 16: A Specific Chromosomal Pathway for the Origin of Human Malignancy?

**DOI:** 10.1038/bjc.1972.7

**Published:** 1972-02

**Authors:** M. A. Bender, M. A. Kastenbaum, Claudia S. Lever

## Abstract

Minkler, Gofman and Tandy (1970a, b) have recently reported data on the karyotype constitutions of human tissue culture cell lines and human tumours, as gathered by a semi-automatic chromosome analysis system. The data appears to show a relationship between the relative number of “number 16” chromosomes and malignancy. We have tested the ability of the “cutting line” approach they used to correctly classify chromosomes from a sample of 723 cells from 100 normal subjects. The cutting line scheme gave very different results from those of an experienced cytogeneticist. The method also failed to give correct average numbers of chromosomes per class. We are thus led to question the conclusions reached by Minkler *et al.* It appears possible that their relatively consistent finding of an excess of “number 16” chromosomes in their largely hyperploid material may be an artefact of their classification scheme, arising from measurement normalization problems, rather than a reflection of a real excess of “number 16” or even of “number 16-like” chromosomes.


*Br. J. Cancer* (1972) **26**, 34.

### CHROMOSOME 16: A SPECIFIC CHROMOSOMAL PATHWAY FOR THE ORIGIN OF HUMAN MALIGNANCY?

M. A. BENDER, M. A. KASTENBAUM AND CLAUDIA S. LEVER

*Department of Radiology, Vanderbilt University, The Tobacco Institute, Inc., and Mathematics Division, Oak Ridge National Laboratory**

Received for publication July 1971

**Summary.—Minkler, Gofman and Tandy (1970a, b) have recently reported data on the karyotype constitutions of human tissue culture cell lines and human tumours, as gathered by a semi-automatic chromosome analysis system. The data appears to show a relationship between the relative number of " number 16 " chromosomes and malignancy. We have tested the ability of the " cutting line " approach they used to correctly classify chromosomes from a sample of 723 cells from 100 normal subjects. The cutting line scheme gave very different results from those of an experienced cytogeneticist. The method also failed to give correct average numbers of chromosomes per class. We are thus led to question the conclusions reached by Minkler *et al.* It appears possible that their relatively consistent finding of an excess of " number 16 " chromosomes in their largely hyperploid material may be an artefact of their classification scheme, arising from measurement normalization problems, rather than a reflection of a real excess of " number 16 " or even of " number 16-like " chromosomes.**

MINKLER *et al.* (1970*a*, b) have reported that " a consistent chromosome abnormality exists in 17 human cell lines and in 11 fresh cancers ". They state that in each of the tissue culture cell lines and in 10 of the 11 cancers they observed a " marked excess of E16 chromosomes per cell, either absolute or in relationship to other chromosome classes ". Minkler *et al.* feel that their observations offer strong support for the hypothesis of Boveri (1914) that an imbalance in cellular chromosome content might destine such cells to malignant behaviour and thus constitutes the origin of neoplasia. However, we feel that there are reasons for questioning this conclusion.

Boveri's hypothesis was based, of course, on the well known tendency of tumour cells to have very abnormal chromosomal constitutions. Boveri himself was never able to resolve the basic question of whether chromosomal abnormalities caused the cells to become neoplastic or whether, on the other hand, becoming neoplastic predisposed cells to mitotic irregularities, thus causing the observed chromosomal abnormalities. The question is still being argued. Koller (1960) in reviewing an extensive literature on the subject concluded: " Variation in chromosome numbers occurs in tumour cells, not because it matters more than in normal cells, but because it matters less ". Nevertheless, the nature of the problem is such that it is still possible to adhere to Boveri's idea, and it must be admitted that a demonstration of any chromosomal *sine qua non* for tumours in even a single species would certainly offer substantial support. Unfortunately, the observations reported by Minkler *et al.* may not constitute such a demonstration.

There are several reasons for our reser-

* Operated by the Union Carbide Corp. for the U.S. Atomic Energy Commission.

vations regarding Minkler *et al.'s* conclusions. All of their chromosome classification was done by means of a semi-automatic karyotyping system [described in a series of University of California Radiation Laboratory reports cited by Minkler *et al.*, 1970*a b* (Gofman, Minkler and Tandy, 1967; Stone, 1967; Stone and Littlepage, 1967; Stone, Littlepage and Clegg, 1969)]. A computer programme was used to classify arm length measurements made by a modified FIDAC film scanner (Ledley, 1964) from stylized tracings of the actual chromosome images. While this system is said by Minkler *et al.* to perform " quantitative karyotyping ", it does not in fact yield results comparable to the subjective karyotyping method in common use by human cytogeneticists. This is not because the authors' semi-automatic method is completely objective (indeed it appears that the tracing process must actually be fairly subjective; for example, the tracer identifies the acrocentric chromosomes as such by simply not drawing any short arm, thus giving the D group, G group and Y chromosomes a centromere index of 0·0). It is rather because what are reported are *average* numbers of chromosomes of each type in a population of cells, instead of the absolute numbers in each individual cell.

Minkler *et al.* (1970*b*, Table III) report that their system gives a mean number of " number 16 " chromosomes in a series of 1834 normal cells of 2·06 $\pm$ 0·031 per cell for males and 2·05 $\pm$ 0·031 for females. But if the estimate of the standard error is 0·031, and the expression

$$s_m = \frac{s}{\sqrt{N-1}},$$

was used to derive the standard error of the mean, $s_m$, the standard deviation, $s$, is greater than 0·9. Thus the average deviations from the means would appear to have been almost one number 16 chromosome per cell!

Second, even for cases (which appear from the standard errors given by Minkler *et al.* to have been rare) where their system found the expected number of chromosomes of each type for a given normal cell, there is no assurance that the same chromosomes would have been assigned to each type or group by a human cytogeneticist. It is possible in such cases that what the authors' system called a number 16 chromosome might have been called a C group chromosome by a cytogeneticist, and that this was compensated for by calling a chromosome the cytogeneticist would have assigned to the C group a number 16 chromosome. It could easily be argued, of course, that the system's assignment may actually have been the correct one, in the genetic sense. There is surely a great deal of uncertainty in the identification of many of the human chromosomes on the basis of length and centromere index. Nevertheless, the standard errors reported by Minkler *et al.* for even the almost certainly identifiable number one and number two pairs are high enough so that serious disagreement with the currently accepted subjective karyotyping methods seems very likely.

Finally, even if the karyotypes generated by Minkler *et al.'s* classification system for normal cells are accepted as correct, there is reason to question whether the system is actually able to deal adequately with cells with the high and variable chromosome numbers characteristic of both the tissue culture cell lines and the tumour cells they analysed. The problem of normalization of length measurements for cells with atypical chromosome numbers is, as noted by Minkler *et al.*, by no means a trivial one. The normalization they used assumes that the " extra " chromosomes in the complement are of an average length corresponding to 1/46 of the average total length of the normal human chromosome set. To the extent that this assumption is not valid, the results will be in error. It is evident that the iterative normalization correction method they describe does not really solve this problem, since it is circular and must perpetuate, rather than correct, any initial large error caused by the original assumption. It is reasonable, then, to ask whether the problem of dealing with cells with highly abnormal chromosome numbers might not have caused a systematic error in the system's chromosome classifications, and thus whether the rather consistent excess of number 16 chromosomes found by Minkler *et al.* might not in fact be an artefact of the system they use for chromosome classification.

A proper test of these questions can in our opinion only be made by testing the classification system on measurements derived from cells also analysed by conventional subjective karyotyping methods. Such a test of performance with respect to currently accepted classification methods is necessary before *any* new classification method can be evaluated, or even understood. Because we happened to already possess a large number of suitable chromosome measurements from normal cells, we have undertaken to test the performance of the Minkler *et al.* scheme against that of a human cytogeneticist. We have not attempted to test the scheme with measurements of chromosomes from tumours or tissue culture cell lines, but the results we have obtained from our normal cell measurements suggest that this would not be very useful in any case.

### MATERIAL AND METHODS

### *Material*

The material used for these tests has been described in detail previously (Bender and Kastenbaum, 1969). In brief, chromosome arm measurements were made for a sample of 723 normal cells from a sample of 100 normal human subjects, using a simple measuring aid consisting, essentially, of a pair of automatic printing dividers (Bender, Davidson and Kastenbaum, 1966). The cells were analysed independently by an experienced human cytogeneticist, and the measurements then assigned to the chromosome pairs on the basis of the cytogeneticist's identifications. It must be emphasized that the identifications were those of a single cytogeneticist, and that other cytogeneticists might have made other assignments, at least among pairs within groups. Nevertheless, there was remarkably good agreement between these assignments and those made independently by other cytogeneticists for a sub-sample of these cells. Furthermore, the two-dimensional vectors of arm length means together with the associated tolerance areas derived from these measurements have been used as the basis for a set of computer programmes for a semi-automatic chromosome analysis system that produces karyotypes acceptable to human cytogeneticists with remarkable success (Bender *et al.*, 1971), giving us added confidence that the assignments for the original sample were as good as current subjective " cut and paste " karyotyping can produce.

### *Methods*

The chromosome analyses of Minkler *et al.* (1970*b*) employed a simple " cutting line " scheme in which the position of each chromosome is located in a two-dimensional space on the basis of total relative length and centromere index. Lines are drawn within the space that cut off areas of various shapes. Chromosome assignments are made simply on the basis of the particular area within which a given length-index point lies. A simple computer programme was written so that we could duplicate this analysis, using Minkler *et al.'s* cutting lines, but without losing track of the original pair assignment of each chromosome in our sample.

A simple comparison of the mean values for centromere index with the cutting line diagram (Minkler *et al.*, 1970*b*, Fig. 1) revealed some obvious problems. First, as already noted, Minkler *et al.'s* acrocentric chromosomes are actually measured as though they were telocentric, with a centromere index of 0·0. Unfortunately, it is not clear from their description of the chromosome tracing step through which this conversion is made whether the tracer draws only the long arm or whether the total of both long and short arms is traced as a single arm. We therefore were forced to make several of our tests in two ways: first, by taking the long arm length as the total length and assigning a centromeric index of zero, and second, by taking the total measured length of both arms and assigning a centromeric index of zero.

A second problem resulted from the fact that the mean value of centromere index for chromosome " number 16 " in our measurement sample (0·367) is quite different from the average value for the sample of Minkler *et al.* (0·44). This puts our mean value below the cutting line at centromere index 0·395 that Minkler *et al.* use to separate the number 16 chromosomes from the number 17 and 18 chromosomes, and results in a large proportion of our " number 16 " chromosomes being classified as number 17 or 18 chromosomes solely on the basis of centromere index. Thus even before any detailed analysis was attempted it was apparent that the cutting line scheme would more often than not misclassify our chromosomes " number 16 " on the basis of centromere index alone.

A final problem was also obvious on inspection of our data in relation to the cutting lines assigned by Minkler *et al.* The centromere index is a dimensionless quantity and thus not affected by the scaling or normalization of chromosome arm lengths. Depending on the scaling factor chosen for normalization, however, the locations of the individual chromosome measurements move laterally in Minkler *et al.'s* cutting line diagram. They can in fact be made to lie within any of the assignment areas of the diagram consistent with their vertical (centromere index) position. Unfortunately, there is no simple scaling factor that will keep all of our own measurements within the lateral limits of the normal chromosome areas in the cutting line diagram. Our total chromosome lengths average considerably less than those of Minkler *et al.* Adjusting our mean total lengths (Bender and Kastenbaum, 1969, Table 5) for total chromosome, rather than total autosome scaling, and normalizing to the average total length given in the cutting line diagram gives a total length of the " number one " chromosome of 12·3, which while still within the diagram, is considerably higher than Minkler *et al.'s* mean of 10·8. At the other end of the diagram, our normalized value for " chromosome 20 " is only 2·2, not only considerably less than Minkler *et al.'s* mean of 3·3 for the F group, but actually falling right upon the left-most cutting line of the diagram. This, of course, results in many of our individual measurements being outside of the space limits. In an attempt to improve this situation, we selected a series of scaling factors that optimized the performance of the cutting line diagram for our data for particular chromosomes, and make our tests on this basis.

### RESULTS

*Average numbers of chromosomes per class.*—The performance of Minkler *et al.'s* cutting line scheme in an analysis of our measurements (adjusted to the same base as their data) is given in Table I. As can be seen, the results are not nearly as good as those reported by Minkler *et al.* (1970*a*,

TABLE I.—*Summary of Chromosome Classification*

| Chromosome class | Males (362 cells) Exp. No. | Mean | S.E. | Females (361 cells) Exp. No. | Mean | S.E. | Total (723 cells) Mean | S.E. |
|---|---|---|---|---|---|---|---|---|
| A1 | 2·0 | 1·754 | 0·042 | 2·0 | 1·701 | 0·042 | 1·728 | 0·030 |
| A2 | 2·0 | 2·702 | 0·054 | 2·0 | 2·634 | 0·051 | 2·668 | 0·037 |
| A3 | 2·0 | 1·345 | 0·042 | 2·0 | 1·399 | 0·044 | 1·372 | 0·030 |
| B | 4·0 | 4·738 | 0·065 | 4·0 | 4·651 | 0·065 | 4·694 | 0·046 |
| C+X | 15·0 | 12·135 | 0·132 | 16·0 | 13·288 | 0·125 | 12·711 | 0·093 |
| D | 6·0 | 5·425 | 0·042 | 6·0 | 5·440 | 0·040 | 5·433 | 0·029 |
| E 16 | 2·0 | 0·453 | 0·036 | 2·0 | 0·432 | 0·035 | 0·443 | 0·025 |
| E (17+18) | 4·0 | 3·102 | 0·084 | 4·0 | 3·330 | 0·085 | 3·216 | 0·060 |
| F | 4·0 | 4·508 | 0·093 | 4·0 | 4·693 | 0·098 | 4·600 | 0·067 |
| G+Y | 5·0 | 4·351 | 0·066 | 4·0 | 3·532 | 0·055 | 3·942 | 0·046 |
| Marker 1 | 0·0 | 0·644 | 0·071 | 0·0 | 0·526 | 0·059 | 0·585 | 0·046 |
| Marker 3 | 0·0 | 0·580 | 0·036 | 0·0 | 0·501 | 0·036 | 0·541 | 0·025 |
| Marker 4 | 0·0 | 1·729 | 0·097 | 0·0 | 1·709 | 0·097 | 1·719 | 0·068 |
| Marker 5 | 0·0 | 0·425 | 0·050 | 0·0 | 0·330 | 0·041 | 0·378 | 0·032 |
| Marker 15 | 0·0 | 2·108 | 0·135 | 0·0 | 1·834 | 0·132 | 1·971 | 0·094 |

Scale factor 0·0.
(Mean = Mean No. of chromosomes/cell; S.E. = Standard error of mean.)
Exp. No. = Expected No. of chromosomes/cell.

TABLE II.—*Summary of Chromosome Classification*

| Chromosome class | Males (362 cells) Exp. No. | Mean | S.E. | Females (361 cells) Exp. No. | Mean | S.E. | Total (723 cells) Mean | S.E. |
|---|---|---|---|---|---|---|---|---|
| A1 | 2·0 | 2·061 | 0·053 | 2·0 | 2·061 | 0·053 | 2·061 | 0·037 |
| A2 | 2·0 | 3·586 | 0·075 | 2·0 | 3·391 | 0·072 | 3·488 | 0·052 |
| A3 | 2·0 | 0·823 | 0·045 | 2·0 | 0·914 | 0·047 | 0·869 | 0·033 |
| B | 4·0 | 6·420 | 0·095 | 4·0 | 6·421 | 0·097 | 6·420 | 0·068 |
| C+X | 15·0 | 9·785 | 0·126 | 16·0 | 11·219 | 0·128 | 10·501 | 0·094 |
| D | 6·0 | 3·688 | 0·075 | 6·0 | 3·291 | 0·069 | 3·490 | 0·051 |
| E 16 | 2·0 | 0·950 | 0·053 | 2·0 | 0·909 | 0·050 | 0·929 | 0·036 |
| E (17+18) | 4·0 | 4·210 | 0·083 | 4·0 | 4·377 | 0·077 | 4·293 | 0·056 |
| F | 4·0 | 3·271 | 0·062 | 4·0 | 3·432 | 0·051 | 3·351 | 0·040 |
| G+Y | 5·0 | 3·975 | 0·055 | 4·0 | 3·524 | 0·043 | 3·750 | 0·036 |
| Marker 1 | 0·0 | 0·133 | 0·026 | 0·0 | 0·116 | 0·027 | 0·124 | 0·019 |
| Marker 3 | 0·0 | 3·204 | 0·064 | 0·0 | 3·069 | 0·063 | 3·137 | 0·045 |
| Marker 4 | 0·0 | 0·381 | 0·060 | 0·0 | 0·338 | 0·058 | 0·360 | 0·042 |
| Marker 5 | 0·0 | 0·914 | 0·061 | 0·0 | 0·737 | 0·054 | 0·826 | 0·041 |
| Marker 15 | 0·0 | 2·599 | 0·155 | 0·0 | 2·202 | 0·149 | 2·401 | 0·108 |

Scale factor 0·80.
(Mean = Mean No. of chromosomes/cell; S.E. = Standard error of mean.)

Table IIIa; 1970*b*, Table III) for their sample of cells from normal individuals. Shifting the data to either the left or right in order to centre various groups within the limits defined by the cutting lines produced no appreciable overall improvement. As an example, Table II gives the results for a shift (Scale Factor) of +0·8, which maximizes the number of " number 16 " assignments.

*Frequency of " correct " assignments.*—Obviously it would be impractical to present here more than a summary of the results of our tests of the frequency with which the cutting line karyotyping scheme made the same assignments as our human cytogeneticist. In brief, as already expected from the distributions of individual chromosome measurement values around the mean values, the cutting line scheme's performance was poor. This is illustrated by Fig. 1, which shows a plot of all the individual measurements for the " number one " and the " number 16 " chromosomes in our 723 cell sample on Minkler *et al.*'s cutting line diagram, but with the total lengths shifted by 1·0 unit to the right so as to maximize the percentage of correct assignments for chromosomes in the G + Y group (for long arms only). This scale factor is not far from optimum for chromosome 16. It will be seen that the maximum of only 29% for correct classification of " number 16 " is so low as it is mainly because so many of the chromosomes have a centromere index below 0·395. There were in fact only 492 " number 16 " chromosomes that had a centromere index above 0·395, but approximately 85% of this subpopulation was correctly classified on the basis of total length. However, selection of the scale factor of +0·8, which optimized the identification of " number 16 " chromosomes to 425/1446 actually decreased the percentage of correct classifications of other chromosomes. Thus, for this scale factor, only about 64% of the " number one " chromosomes were correctly classified. This was raised to a maximum of 73% by application of a scale factor of —0·5, but when this was done the percentage of " number 16 " chromosomes correctly identified dropped to only a little more than 3%. Similar relationships were, of course, observed for all of the other chromosomes as well.

As already pointed out, the acrocentric chromosomes presented a special problem for our tests both because a centromere index of 0·0 is required by the cutting line diagram and because of our uncertainty about whether long arm lengths or total lengths were traced for Minkler *et al.*'s

Fig. 1.—Plot of chromosomes number 1 and number 16 from 723 normal human cells. The cutting line diagram is that of Minkler *et al.* (1970*a*, *b*). The length measurements were normalized to Gofman *et al.*'s average total chromosomal length and then shifted 1·0 units to the right in order to centre the number 16 chromosome points approximately on the " E 16 " area.

sample. Table III shows the distributions of chromosomes identified by the cytogeneticist as belonging to the G + Y group with respect to the limits of the G + Y line on the cutting line diagram. A scaling factor of +1·0 produced the largest number correctly classified in the case where the long arm length only was used, while a scale factor of + 0·3 was necessary when total length was used. Neither of these factors is optimal for the " number 16 " chromosomes, however. As already noted, the + 1·0 value lowers the percentage of successful classifications slightly (0·4%) and the + 0·3 value decreases it still further to 21%.

TABLE III.—*Distributions of G + Y Group Total Lengths*

| | Length interval | | |
|---|---|---|---|
| Scaling | < 1·3 | 1·3–3·1 | > 3·1 |
| $X_L$ . . | 1659 | 1593 | 1 |
| $X_L$+0·3 . | 704 | 2542 | 7 |
| $X_L$+1·0 . | 57 | 3082 | 114 |
| $X_T$ . . | 432 | 2794 | 27 |
| $X_T$+0·3 . | 266 | 2899 | 88 |
| $X_T$+1·0 . | 37 | 2370 | 846 |

($X_L$ = length of long arm only; $X_T$ = sum of long and short arms.)

### DISCUSSION

Though the cutting line scheme used by Minkler *et al.* gave average numbers of chromosomes per class which are not statistically different from those expected for normal cells when used to analyse their own set of measurements, it clearly does not do so when used to analyse our set of normal human chromosome measurements. We cannot explain this difference. Clearly, however, there is no simple transformation of our raw measurements that can make the average chromosome numbers in all classes close to the expected numbers. Whether this would be so for other sets of measurements of normal human chromosome sets remains a matter of speculation, though the mean values for centromere index of chromosome " number 16 " from two other reported measurement sets (Penrose, 1964; Turpin and Lejeune, 1965) are both about 0·4, leading one to suspect that the frequency with which the cutting line scheme would correctly classify this chromosome would be quite low.

In addition, it is apparent that neither the scheme of Minkler *et al.* nor for that matter *any* simple two-dimensional linear discrimination scheme can be used to produce individual karyotype analyses that are acceptable to the human cytogeneticist. The variability of arm lengths between cells and even between pairmates is simply too great. It is thus clear that even in their sample of cells from normal people the chromosomes classified by Minkler *et al.'s* system as number 16 chromosomes were frequently *not* number 16 chromosomes, either in the strictly genetic sense or in the morphological sense that at least one human cytogeneticist would agree with their assignments.

Minkler *et al.* (1970*b*) have argued that whatever one wishes to label them, " chromosomes of constant dimensional characteristics and normalized to account for differential contraction consistently appear as a possible specific common chromosomal pathway for the origin of human malignancy ". Our reason for emphasizing the impossibility of selecting a single scale factor or normalization that would optimize the performance of the cutting line scheme for the analysis of our normal human chromosome measurements was that it suggests a simple explanation for the relatively consistent result obtained by Minkler *et al.* Virtually all of the cells they examined from both the tissue culture cell lines and the tumours were characterized by hyperploidy. Both chromosomal variability and rapid change in stem line chromosomal constitutions in response to external influences are well known in such cells. These phenomena suggest that the proportions of chromosomes of various types in the hyperploid cells are unlikely to be the same as those of the normal dioloid, a circumstance also already well known.

Furthermore, as recently noted by Muldal, Elejalde and Harvey (1971), random chromosomal breakage is expected to change the distribution of chromosomes in the various morphological classes. Breakage will most often simply reduce the length of one arm of a chromosome, and because it occurs more often in longer chromosomes will tend to skew the distributions of chromosomes in the various classes in the direction of shorter lengths. Because most human chromosomes are submetacentric or acrocentric, random breakage will also skew the distribution of centromeric index toward higher values. In other words, random breakage is expected to push the distribution of correctly normalized measurements in the general direction of the number 16 area in Minkler *et al.'s* cutting line diagram. Indeed, this is exactly the observation reported by Muldal *et al.* (1971).

Not only does all this lead one to expect that cell lines and tumours might contain a disproportionate number of morphologically number 16-like chromosomes, but it also makes unlikely the assumption that the chromosomes of such hyperploid cells have the same average lengths as the average length of the normal human chromosome set. To the extent the assumption is in error, the chromosome measurements will of course be shifted to the right or the left in the cutting line diagram. Though a detailed evaluation of the effects of such shifts on the distributions of chromosomes among classes as determined by the cutting line diagram is not possible in the absence of information on the means and distributions of lengths for individual chromosome types in Minkler *et al.'s* sample, simple inspection of their mean values in relation to their cutting line diagram will give a general idea of what must happen.

From Fig. 2, it can be seen that the interval of lengths that defines the F group in Minkler *et al.'s* diagram lies from 2·20 to 3·81 at centromere index 0·45 (the mean given for the F group), but the mean length for the F group is given as 3·3. In other words, only 0·51 length units separate an average F group chromosome in Minkler *et al.'s* sample from the cutting line dividing the F group and the number 16 chromosome areas. The length interval for the number 16 chromosomes runs from 3·78 to 5·35 at the mean centromere index of 0·44. In this case, however, the mean value for length is nearer the middle of the space at 4·3, 0·52 units from the left-hand cutting line and 1·05 units from the right-hand line. Thus as illustrated by Fig. 2, a scaling error that makes all chromosomes too long by 0·7 unit causes all of the real F group chromosomes *plus all of the "real" number 16 chromosomes* to be classified as number 16 chromosomes, provided only that they are all of average dimensions! In such a case the ratio of "number 16" chromosomes would certainly be significantly elevated, not because there were

FIG. 2.—Mean chromosome measurements of Minkler *et al.* plotted with and without an arbitrary scaling error. ● Mean values from Minkler *et al.* (1970*b*), Table I(b). ▲ Same values shifted 0·7 unit to simulate a length normalization error.

really extra number 16 chromosomes, but simply because the average length of a chromosome in the cell was smaller than 1/46 of the average total length in a normal diploid cell. A similar consideration of the means for other chromosome classes in relation to the cutting lines shows that the classification of other chromosome types is relatively much less sensitive to scaling error. A shift of 0·7 to the right does not, for example, cause an average length chromosome of any of the other classes to be misclassified!

Obviously this is an extreme example. Real chromosomes will rarely be of average dimensions, and the error caused by the scaling assumption is likely to be less than 0·7 units. But it illustrates that there is the possibility of a built-in, systematic classification error in Minkler *et al.*'s cutting line scheme. The artefact results basically from the fact that the space defining the " number 16 " class is narrower than that for any other chromosome class, plus the asymmetrical locations of the mean values for the F group and number 1 chromosome lengths in their spaces (this latter factor is, of course, what keeps scaling errors in either direction from greatly increasing the frequency of " marker 4 " or " marker 5 " chromosomes).

Faced with the results of our tests of the performance of the cutting line chromosome analysis scheme with our set of normal chromosome measurements, and with the possibility of a systematic artefact of the cutting line method that would be expected to most consistently increase the relative frequency of chromosomes classified as number 16 chromosomes in relation to other classes, we are forced to conclude that no common chromosomal pathway for the origin of human malignancy has yet been demonstrated.

We are grateful to Norma C. Hull and Kim O. Bowman for programming assistance and to Dr Howard I. Adler for his support. This work was supported by the U.S. Atomic Energy Commission.

### REFERENCES

BENDER, M. A. & KASTENBAUM, M. A. (1969) Statistical Analysis of the Normal Human Karotype. *Am. J. hum. Genet.*, **21**, 322.

BENDER, M. A., DAVIDSON, J. B. & KASTENBAUM, M. A. (1966) Chromosome Analysis. In *Use of Computers in Analysis of Experimental Data and Control of Nuclear Facilities.* U.S. Atomic Energy Commission CONF-660527. p. 121.

BENDER, M. A., KASTENBAUM, M. A., LEVER, C. S. & PELSTER, D. R. (1971) *Computers in Biology and Medicine* (in press).

BOVERI, T. H. (1914) *Zur Frage der Entstehung maligner Tumoren.* Jena: Gustav Fischer.

GOFMAN, J. W., MINKLER, J. L. & TANDY, R. K. (1967) *A Specific Common Chromosomal Pathway for the Origin of Human Malignancy.* University of California: Lawrence Radiation Laboratory Reports (UCRL-50356).

KOLLER, P. C. (1960) Chromosome Behavior in Tumors: Readjustments to Bovari's Theory. *Cell Physiology of Neoplasia.* Austin: Univ. of Texas Press.

LEDLEY, R. S. (1964) High-speed Automatic Analysis of Biomedical Pictures. *Science, N.Y.*, **146**, 216.

MINKLER, J. L., GOFMAN, J. W. & TANDY, R. K. (1970*a*) A Specific Common Chromosomal Pathway for the Origin of Human Malignancy II. *Adv. biol. med. Phys.*, **13**, 108.

MINKLER, J. L., GOFMAN, J. W. & TANDY, R. K. (1970*b*) A Specific Common Chromosomal Pathway for the Origin of Human Malignancy II. *Br. J. Cancer*, **24**, 726.

MULDAL, S., ELEJALDE, R. & HARVEY, P. W. (1971) Specific Chromosome Anomaly Associated with Autonomous and Cancerous Development in Man. *Nature, Lond.*, **229**, 48.

PENROSE, L. L. (1964) A Note on the Mean Measurements of Human Chromosomes. *Ann. hum. Genet.*, **28**, 195.

STONE, S. P. (1967) *Chromosomal Scanning Program at LRL.* Part I. *A Set of Chromosome Pattern-Recognition Programs.* University of California; Lawrence Radiation Laboratory Reports (UCRL-50364, Part I).

STONE, S. P. & LITTLEPAGE, J. L. (1967) *The Chromosome Scanning Program at Lawrence Radiation Laboratory.* University of California: Lawrence Radiation Laboratory Reports (UCRL-70413).

STONE, S. P., LITTLEPAGE, J. L. & CLEGG, B. R. (1969) *Second Report on the Chromosome Scanning Program at the Lawrence Radiation Laboratory.* University of California: Lawrence Radiation Laboratory Reports (UCRL-71493).

TURPIN, R. & LEJEUNE, J. (1965) *Les chromosomes humains.* Paris: Gauthier-Villars.